# Endothelial PHD2 deficiency induces apoptosis resistance and inflammation via AKT activation and AIP1 loss independent of HIF2α

**DOI:** 10.1152/ajplung.00077.2024

**Published:** 2024-08-19

**Authors:** Shuibang Wang, Keytam S. Awad, Li-Yuan Chen, Mohammad A. H. Siddique, Gabriela A. Ferreyra, Caroline L. Wang, Thea Joseph, Zu-Xi Yu, Kazuyo Takeda, Cumhur Y. Demirkale, You-Yang Zhao, Jason M. Elinoff, Robert L. Danner

**Affiliations:** ^1^Critical Care Medicine Department, NIH Clinical Center, National Institutes of Health, Bethesda, Maryland, United States; ^2^National Heart Lung and Blood Institute, National Institutes of Health, Bethesda, Maryland, United States; ^3^Section for Injury Repair and Regeneration, Stanley Manne Children Research Institute, Ann & Robert H. Lurie Children’s Hospital of Chicago, Chicago, Illinois, United States; ^4^Department of Pediatrics, Northwestern University Feinberg School of Medicine, Chicago, Illinois, United States

**Keywords:** AIP1, AKT, apoptosis, PHD2, STAT

## Abstract

In hypoxic and pseudohypoxic rodent models of pulmonary hypertension (PH), hypoxia-inducible factor (HIF) inhibition attenuates disease initiation. However, HIF activation alone, due to genetic alterations or use of inhibitors of prolyl hydroxylase domain (PHD) enzymes, has not been definitively shown to cause PH in humans, indicating the involvement of other mechanisms. Given the association between endothelial cell dysfunction and PH, the effects of pseudohypoxia and its underlying pathways were investigated in primary human lung endothelial cells. *PHD2* silencing or inhibition, while activating HIF2α, induced apoptosis-resistance and IFN/STAT activation in endothelial cells, independent of HIF signaling. Mechanistically, PHD2 deficiency activated AKT and ERK, inhibited JNK, and reduced AIP1 (ASK1-interacting protein 1), all independent of HIF2α. Like PHD2, *AIP1* silencing affected these same kinase pathways and produced a similar dysfunctional endothelial cell phenotype, which was partially reversed by AKT inhibition. Consistent with these in vitro findings, AIP1 protein levels in lung endothelial cells were decreased in *Tie2-Cre*/*Phd2* knockout mice compared with wild-type controls. Lung vascular endothelial cells from patients with pulmonary arterial hypertension (PAH) showed IFN/STAT activation. Lung tissue from both SU5416/hypoxia PAH rats and patients with PAH all showed AKT activation and dysregulated AIP1 expression. In conclusion, PHD2 deficiency in lung vascular endothelial cells drives an apoptosis-resistant and inflammatory phenotype, mediated by AKT activation and AIP1 loss independent of HIF signaling. Targeting these pathways, including PHD2, AKT, and AIP1, holds the potential for developing new treatments for endothelial dysfunction in PH.

**NEW & NOTEWORTHY** HIF activation alone does not conclusively lead to human PH, suggesting that HIF-independent signaling may also contribute to hypoxia-induced PH. This study demonstrated that *PHD2* silencing-induced pseudohypoxia in human lung endothelial cells suppresses apoptosis and activates STAT, effects that persist despite HIF2α inhibition or knockdown and are attributed to AKT and ERK activation, JNK inhibition, and AIP1 loss. These findings align with observations in lung endothelial cells and tissues from PAH rodent models and patients.

## Graphical abstract

**Figure:**
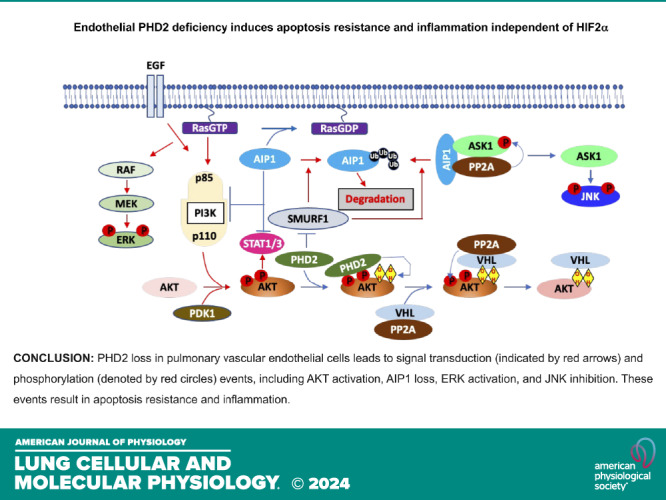

## INTRODUCTION

Hypoxia impairs pulmonary endothelial cell (EC) homeostasis contributing to pulmonary hypertension (PH) and ultimately pulmonary vascular remodeling. Although the role of hypoxia-inducible factor (HIF) signaling, activated by hypoxia itself or a pseudohypoxic state, has been studied in PH, its importance in pathogenesis and value as a monotherapeutic target remains incompletely understood. Prolyl hydroxylase domain (PHD) proteins are oxygen-dependent dioxygenases that regulate oxygen responsiveness by hydroxylating HIF proteins, targeting them for proteasomal degradation via von Hippel–Lindau (VHL)-mediated ubiquitination (1, 2). In mice, PH induced by chronic hypoxia or pulmonary arterial hypertension (PAH) caused by *Tie2*-mediated *Phd2* (*Egln1*, official gene symbol) disruption can be prevented by global or cell-specific deletion or heterozygous knockout of *Hif2α* (*Epas1,* official gene symbol; 3–7). An HIF2α gain-of-function mutation (G537R/W), which protects it from PHD2-mediated hydroxylation, causes autosomal dominant erythrocytosis. PAH has been reported in two of seven affected family members (8), whereas PH has been diagnosed in two additional cases (9). Further supporting the singular importance of HIF2α, mice bearing this mutation also develop PH (8, 10). In addition, patients with autosomal recessive Chuvash polycythemia caused by a VHL inactivating mutation (R200W) are susceptible to PH but are also prone to pulmonary embolism (11–13), a separate potential contributor to elevated pulmonary pressures. Nonetheless, polycythemia and PH in murine models of Chuvash have been prevented by heterozygous deletion or therapeutic inhibition of HIF2α alone (11, 14).

Although the previous evidence strongly implicates HIF signaling and HIF2α specifically in the development of PH or PAH, other work suggests a more complex relationship between hypoxic/pseudohypoxic mechanisms and PH that warrants further investigation. For example, HIF1α activation in pulmonary artery smooth muscle cells was shown to conversely lower vascular tone and protect mice against PH (15). *Cdh5-Cre*-mediated *Hif2α* deletion, rather than overexpression, was found to promote PH (16). Furthermore, daprodustat, an oral PHD inhibitor developed for the treatment of anemia in end-stage renal disease, stabilizes HIF1α and HIF2α, but so far, has not been associated with the development of PH (17, 18). Importantly, PHDs and VHL regulate other non-HIF targets, such as AKT (19) and the B55α subunit of VHL-associated protein phosphatase 2 A (PP2A; 20). Proteins like AKT sit in consequential signaling networks that regulate proliferation, apoptosis, inflammation, cell migration, and cytoskeletal architecture processes highly relevant to pathologic vascular remodeling in PAH (21–23).

Here, disruption of the PHD2/VHL pathway and the role of HIF2α was investigated in a pseudohypoxia model of endothelial dysfunction simulating aspects of PAH and PH pathobiology. *PHD2* silencing produced an apoptosis-resistant and inflammatory cellular phenotype in human lung microvascular endothelial cells (LMVECs) and pulmonary arterial endothelial cells (PAECs). HIF2α protein was stabilized, accompanied by HIF-induced expression of glycolytic genes, but did not fully account for the phenotypic changes or aberrant signaling in these altered cells. AKT, which is regulated by PHD2/VHL independent of HIF2α (19), and AIP1 (ASK1-interacting protein 1, official symbol *DAB2IP*), a Ras GTPase-activating (GAP) scaffolding protein that regulates a number of signaling pathways including AKT, ERK, JNK, and STAT (24), were investigated to determine the mechanistic underpinnings of pseudohypoxic endothelial dysfunction. Finally, lung tissues from the SU5416/hypoxia (SuHx) rat and *Phd2* (*Egln1*) knockout mouse models of PAH, as well as from patients with PAH, were examined to further test the validity of our findings.

## MATERIALS AND METHODS

### Human Primary Lung Endothelial Cell Culture

Human primary lung microvascular endothelial cells (LMVECs) and pulmonary artery endothelial cells (PAECs) from different donors were purchased from Lonza (Walkersville, MD; see Supplemental Table S1, *A* and *B*, for donor information: https://doi.org/10.6084/m9.figshare.26306104). Cells were seeded onto plates and cultured in a complete medium comprising endothelial basal medium-2 (EBM-2) and supplement EGM-2 MV for LMVECs or EGM for PAECs. The culture plates for PAECs were coated with type I collagen (50 μg/mL in 0.02 N acetic acid) prior to cell seeding. The basal medium EBM-2 and supplements were obtained from Lonza. Cells were passaged and used at passages 1–5 for experiments. LMVECs isolated from congenital heart disease (shunt)-associated PAH (APAH) patients and failed donors (FD) were obtained from the Pulmonary Hypertension Breakthrough Initiative (PHBI, see Supplemental Table S1*C*). Lung tissue from idiopathic PAH (IPAH) patients and FD controls, embedded in optimal cutting temperature compound, were also obtained from PHBI (see Supplemental Table S1*D*).

For measurement of mitochondrial mass and superoxide, LMVECs were plated at 0.8 × 10^5^ cells/well in 6-well plates and cultured for 16 h in a complete medium, followed by transfection with specific *PHD2* or control siRNA (15 nM). Seventy-two hours posttransfection, cells were detached using 0.25% trypsin-EDTA (Thermo Fisher Scientific), washed and resuspended with PBS, and incubated for 30 min at 37°C with 100 nM MitoTracker Green FM (Thermo Fisher Scientific) to stain mitochondria, and 3 μM MitoSOX Red (Thermo Fisher Scientific) to detect mitochondrial superoxide. Cells were then labeled with Live/Dead dye and analyzed by MACSQuant Analyzer 10 Flow Cytometer (Miltenyi Biotec, San Diego, CA).

### siRNA Gene Silencing and Quantitative Real-Time PCR

*PHD2*, *AIP1*, *HIF2α*, *HIF1β*, and *SMURF1* were knocked down in cells using 15 nM siRNA each, except for *SMURF1* (30 nM). Specific siRNAs (ON-TARGET plus SMARTpool) and nontargeting control were obtained from Dharmacon (Lafayette, CO; see Supplemental Table S2*A*). Transfection of siRNA into cells was performed by using DharmaFECT1 transfecting reagent (Dharmacon) as per the manufacturer’s protocol.

For quantitative real-time PCR (qPCR), total RNA was extracted using RNeasy kits (Qiagen, Valencia, CA) and quantified by NanoDrop (BioLabNet, VA). cDNA synthesis was then performed using reverse transcription kits (Bio-Rad Laboratories, Hercules, CA). qPCR was carried out with TaqMan probe/primers (Applied Biosystems; see Supplemental Table S2*B* for unique assay information). Beta-actin (*ACTB*), β-2 microglobulin (*B2M*), and ribosomal protein L13a (*RPL13A*) served as internal reference genes, and results were presented as geometric means ± geometric SD.

### Western Blotting and Immunofluorescence

For Western blotting, whole cell lysates or rat lung homogenates were prepared on ice and protein concentrations were determined. Equal amounts of protein were loaded onto NuPAGE Bis-Tris Protein Gels (Thermo Fisher Scientific), following previously described procedures (25). For protein level normalization, Western blots were probed with a horseradish peroxidase-conjugated antibody to beta-actin (Sigma, St. Louis, MO). Quantification of protein bands by densitometry was performed with Image Lab software, version 6.0.1 (Bio-Rad Laboratories). Uncropped Western blots are shown in Supplemental Fig. S6.

For immunofluorescence (IF) staining, lung tissue sections from IPAH patients and FD controls, embedded in optimal cutting temperature compound, were air-dried, fixed with 4% paraformaldehyde at room temperature for 7 min, blocked with 10% donkey serum/PBS and incubated with sheep anti-von Willebrand factor and rabbit anti-pAKT (S-473), anti-PHD2, or anti-AIP1 antibodies overnight at 4°C. Sections were then incubated with Alexa Fluor 488 donkey anti-sheep and Alexa Fluor 594 donkey anti-rabbit IgG (Jackson ImmunoResearch Laboratories; both at 1:200 dilution) at room temperature for 1 h. Nuclei were counterstained with Hoechst 33342 (Thermo Fisher Scientific; 1:5,000) for 15 min at room temperature and mounted with Fluoromount-G (SouthernBiotech, Birmingham, AL) for confocal microscopy. Fluorescence images were acquired using an SP8 DMI6000 confocal microscope (Leica Microsystems, Germany) and quantified using ImageJ (version 1.54i). Small pulmonary vessels were examined in each biological replicate. ECs were identified by staining for von Willebrand factor (vWF; green), an EC marker. The signal intensities of PHD2 and pAKT (both red) in ECs were quantified and normalized to vWF staining. AIP1 (red), expressed in ECs and lung vascular smooth muscle cells (VSMCs), was quantified for ECs using JACop, an ImageJ plugin, to measure colocalization with vWF. Information on antibodies used in this study can be found in Supplemental Table S3.

### Cell Apoptosis Assay

Forty-eight hours posttransfection with specific siRNAs or controls, cells were seeded at 5 × 10^3^ cells/well in 96-well plates and allowed to adhere for 6 h in a complete medium (100 μL/well). The medium was then replaced with fresh complete medium or serum- and growth factor-free medium; specified inhibitors or vehicle control were added to the cell culture medium. Twenty-four hours later, caspase-3/7 activities in cells were measured using a luminometric assay kit Caspase-Glo 3/7 (Promega, Madison, WI). The amount of luminescence was measured on the Victor3 multilabel reader (PerkinElmer Inc.).

Apoptosis was also evaluated by annexin v/propidium iodide (AV/PI) staining. Cells were plated at 0.8 × 10^5^ cells/well in 6-well plates and cultured for 16 h in a complete medium (2 mL/well). Cells were transfected with specific or control siRNAs for 48 h. The medium was then replaced with a fresh complete medium or medium without serum and growth factors; specified inhibitors or vehicle control were added to the cell culture medium. Twenty-four hours later, cells were detached by 0.25% trypsin-EDTA (Thermo Fisher Scientific), washed, and stained using the FITC-Annexin V Apoptosis Detection Kit (BD PharMingen, Franklin Lakers, NJ) as described in the manufacturer’s protocol. Apoptotic cells (AV+/PI−) were gated and counted using the MACSQuant Analyzer 10 Flow Cytometer (Miltenyi Biotec).

### Rodent Models of PAH

The SU5416/hypoxia PAH rat model (referred as SuHx rats) was established as originally described (26). Briefly, Sprague-Dawley rats (Charles River Laboratories, Wilmington, MA) were subcutaneously injected with SU5416 (25 mg/kg; Tocris, Minneapolis, MN), exposed to hypoxia (10% FiO2) for 3 wk in a Biospherix Oxycycler (Biospherix Ltd., Parish, NY) and then returned to normoxia for 7 wk. At *week 3* and *week 8*, lung tissue samples were collected and homogenized for Western blotting. All aspects of animal testing and care were approved by the Animal Care and Use Committee at the National Institutes of Health Clinical Center.

*Egln1^Tie2^*(*Phd2*CKO) PAH mice were established, as previously described (5). The *Phd2*CKO mice and their control wild-type (WT) littermates are all on a C57BL/6J background. Male and female mice of both genotypes (WT and *Phd2*CKO) were used at 3 mo of age. Paraffin-embedded lung tissue sections (5 µm thick) of the PAH mice and their WT littermates were sent to Histoserv, Inc. (Germantown, MD) for immunohistochemistry (IHC) using anti-AIP1 as the first antibody, HRP-conjugated anti-rabbit as secondary antibody, and DAB (3,3′-diaminobenzidine) as a chromogen substrate. Images were analyzed and quantified using ImageJ (version 1.54i, Color deconvolution2 plugin). Multiple small pulmonary vessels from each biological replicate were examined, and the signal intensity of AIP1 was normalized to the hematoxylin staining of cell nuclei.

### Microarray Experiments and Analysis

Microarray analysis was conducted on samples from five independent experiments using LMVECs from the same donor at the same passage number to minimize variability. Cells were transfected with either nontargeting control siRNA (siCTRL), *PHD2* (siPHD2, 15 nM), *AIP1* (siAIP1, 15 nM), or both (siBoth) siRNA for 48 h, and total RNA was then extracted using RNeasy miniprep kits (Qiagen). RNA quantity and quality were assessed using the NanoDrop 8000 Spectrophotometer (Thermo Fisher Scientific) and RNA 6000 Nano LabChip (Agilent 2100 Bioanalyzer, Santa Clara, CA), respectively. All samples exhibited intact 18S and 28S ribosomal RNA bands (RIN 8.6–10, 260/280 ratios 2.00–2.04) and underwent microarray expression analysis using the Human Clariom S Assay (Thermo Fisher Scientific). Microarray data were RMA-normalized and log2-transformed in Thermo Fisher Transcriptome Analysis Console, annotated with Human Clariom S Assay, and imported into R for principal component analysis (PCA). Both raw and normalized data were submitted to GEO (GSE250030) and complied with MIAME. Limma fitted linear mixed models with fixed effects (siPHD2, siAIP1, and their interaction) and a random effect (replication) to expression data for each gene. Differentially regulated genes were identified using a false discovery rate (FDR) of 0.01, a 1.5-fold change cutoff for siPHD2 and siAIP1 main effects, and an FDR of 0.1 for interaction effects. This resulted in 1,728 unique differentially regulated genes, of which 853 were regulated by siPHD2, 681 by siAIP1, and 928 by interaction, with overlap among the groups. These groups were then uploaded for ingenuity pathway analysis (IPA).

### Quantification and Statistical Analysis

Differences in relevant gene expression (mRNA and protein), protein phosphorylation, MitoSOX, and MitoTracker between control and PHD2 siRNA-transfected human LMVECs or PAECs were analyzed by paired *t* test. Differences between lung tissues or cells from controls and rodent PAH models or from patients with PAH and non-PAH subjects were examined by unpaired *t* test. Dose-dependent effects of dimethyloxalylglycine (DMOG) on caspase3/7 activities, protein levels, and protein phosphorylation were tested by fitting linear regression models with LMVEC or PAEC donors as random effects. ANOVA followed by the post hoc *t* test was used to analyze effects of gene silencing on cell apoptosis, protein phosphorylation, gene expression, and cytokines levels in the presence and absence of various siRNA and pathway inhibitors. Statistical analysis was performed using JMP version 16 (SAS Institute) with a two-tailed α of 0.05 as the cut-off for significance. Data are represented as arithmetic means ± SD except mRNA levels measured by qPCR, for which geometric means of fold changes (FCs) were plotted.

## RESULTS

### *PHD2* (*Egln1*) Silencing Induces Glycolytic Genes and Apoptosis Resistance in LMVECs

**Figure 1.**
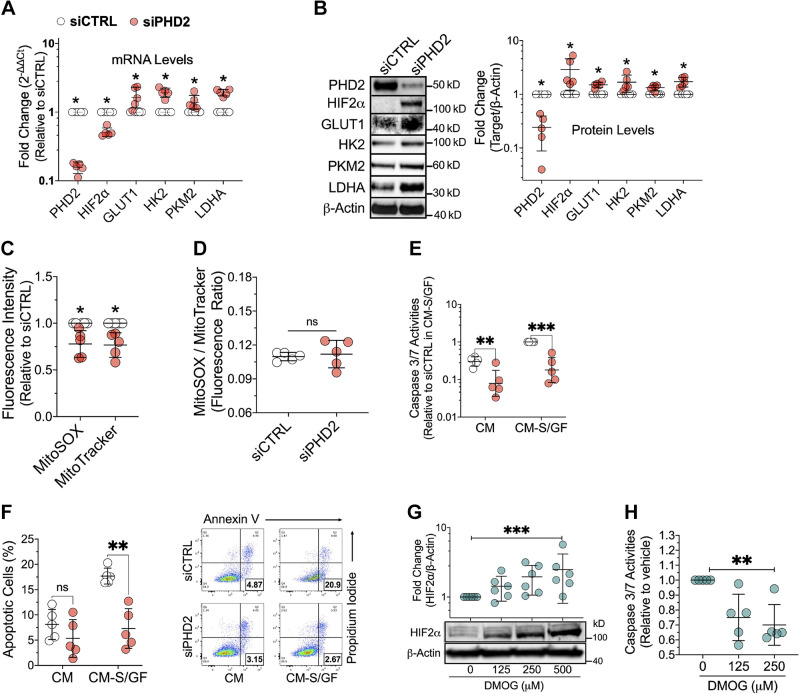
*PHD2* silencing or inhibition induces glycolytic gene expression and apoptosis resistance in human lung microvascular endothelial cells. *A*: mRNA levels in cells transfected with control (siCTRL) or *PHD2* (siPHD2) siRNA for 48 h were measured by qPCR (*n* = 5). Data shown as geometric means ± geometric SD. *B–D*: protein levels were measured by Western blotting at 72 h posttransfection (*n* = 5). Mitochondrial superoxide and mass were analyzed by flow cytometry at the same time point (*n* = 5). *E* and *F*: following 48 h transfection, cells were incubated in either complete medium (CM) or serum- and growth factor-free medium (CM-S/GF) for 24 h. Apoptosis was assessed by caspase 3/7 assay and AV/PI staining (*n* = 5). *G*: HIF2α protein levels in cells cultured in CM were evaluated by Western blotting after 24 h treatment with DMOG, a PHD2 inhibitor (*n* = 6). *H*: caspase 3/7 activity was measured in CM-S/GF treated cells following 24 h DMOG treatment (*n* = 5). Western blot protein levels were normalized to β-actin. Data are presented as means ± SD. *P < 0.05, **P ≤ 0.01, and ***P ≤ 0.001. Paired *t* test (*A–D*), two-way ANOVA with post hoc *t* test (*E* and *F*), linear regression (*G* and *H*). AV/PI, annexin v/propidium iodide; DMOG, dimethyloxalylglycine; qPCR, quantitative real-time PCR.

To determine the effect of PHD2 deficiency on HIF and HIF-regulated glycolytic genes in human LMVECs, *PHD2* was knocked down using a targeted siRNA approach (Fig. 1, *A* and *B*). As expected, HIF2α protein, the major HIF isoform expressed in ECs, increased in *PHD2*-silenced LMVECs compared with control siRNA (siCTRL) transfected cells while *HIF2α* mRNA was reduced (Fig. 1, *A* and *B*). HIF2α protein stabilization in *PHD2*-silenced LMVECs, as expected, was accompanied by increased mRNA and protein expression of glycolytic genes *GLUT1*, *HK2*, *PKM2*, and *LDHA* (Fig. 1, *A* and *B*), and elevated extracellular lactate levels (Supplemental Fig. S1*A*). Consistent with previous work that demonstrated a decline in both mitochondrial reactive oxygen species and mitochondria DNA content in PHD2-deficient cells (27, 28), mitochondria-derived superoxide (MitoSOX) and mitochondrial mass (MitoTracker) were both decreased in *PHD2*-silenced LMVECs (Fig. 1*C*) while the ratio of MitoSOX to MitoTracker was unaffected (Fig. 1*D*).

Aberrant vascular remodeling in PAH has been associated with apoptosis-resistance and reduced PHD2 expression (5, 29, 30). Here, decreased apoptosis in *PHD2*-silenced endothelial cells was confirmed by caspase 3/7 assay (Fig. 1*E*) and annexin v/propidium iodide (AV/PI) staining (Fig. 1*F*). *PHD2* siRNA reduced caspase 3/7 activation under both complete media (CM) and serum and growth factor withdrawal (CM-S/GF, an apoptosis stimulus) conditions. However, percentages of apoptotic cells measured by AV/PI staining were only significantly suppressed after serum and growth factor withdrawal challenge. Reduced caspase 3/7 activation by PHD2 silencing was further demonstrated by decreased caspase 3 cleavage (Supplemental Fig. S1*B*). Like *PHD2* silencing, dimethyloxalylglycine (DMOG), a cell-permeable PHD2 inhibitor, dose-dependently stabilized HIF2α protein and reduced caspase 3/7 activity (Fig. 1, *G* and *H*).

### Apoptosis Resistance Independent of HIF2α in *PHD2*-Silenced LMVECs

**Figure 2.**
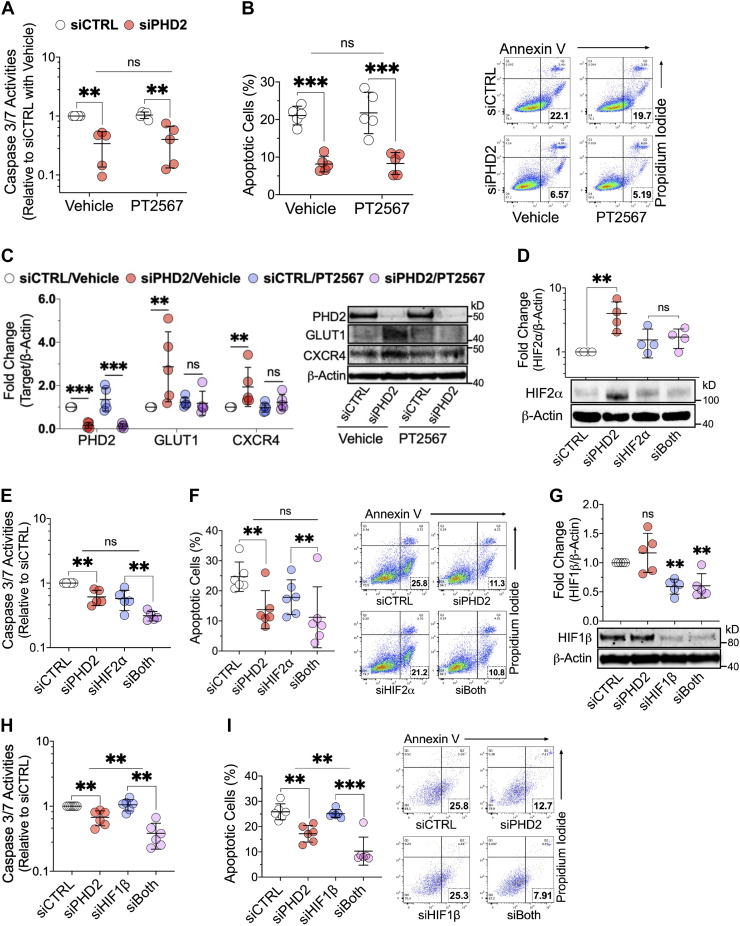
Neither HIF2α inhibition nor silencing reverses apoptosis resistance induced by *PHD2* silencing in human lung microvascular endothelial cells. Cells were transfected with siRNAs (siCTRL, siPHD2, siHIF2α, and siHIF1β) or their combinations for 48 h in complete media (CM). *A* and *B*: following 24 h incubation in serum- and growth factor-free media (CM-S/GF) with PT2567 (HIF2α inhibitor) or vehicle, apoptosis was assessed by caspase 3/7 and AV/PI staining (*n* = 5). *C*: cells were further incubated for 24 h in CM with PT2567 or vehicle for Western blotting (*n* = 5). *D*: Western blotting for cells 48 h posttransfection (*n* = 4). *E* and *F*: following 24 h incubation in CM-S/GF, apoptosis was assessed by caspase 3/7 and AV/PI staining (*n* = 5 or 6). *G*: Western blotting for cells 48 h posttransfection (*n* = 5). *H* and *I*: cells were incubated and tested for apoptosis as in (*E*) and (*F*), respectively (*n* = 6). Western blot protein levels were normalized to β-actin. Data are presented as means ± SD. **P ≤ 0.01 and ***P ≤ 0.001. Two-way ANOVA with post hoc *t* test. AV/PI, annexin v/propidium iodide.

Genetic deletion of *Hif2α* but not *Hif1α* prevents PAH and pulmonary vascular remodeling in *Tie2-Cre*-mediated *Phd2* knockout mice (5, 31). Therefore, PT2567, a specific HIF2α inhibitor, and HIF2α siRNA (siHIF2α) were used to investigate whether the aberrant phenotype of *PHD2*-silenced ECs was entirely dependent on HIF2α. Surprisingly, PT2567 did not affect apoptosis in either *PHD2*-silenced LMVECs (Fig. 2*A*) or *PHD2*-silenced PAECs (Supplemental Fig. S2*A*) as assessed by caspase 3/7 activation and AV/PI staining (Fig. 2*B*), although it effectively blocked expression of known endothelial HIF2α targets GLUT1 and CXCR4 (Fig. 2*C*). Unexpectedly, *HIF2α* knockdown, which blocked the increase in HIF2α protein caused by PHD2 silencing (Fig. 2*D*), did not prevent but rather enhanced apoptosis resistance, as evaluated by caspase 3/7 activation (*P* < 0.001 for the main effect of siHIF2α; Fig. 2*E*) and AV/PI staining (*P* = 0.006 for the main effect of siHIF2α; Fig. 2*F*) in LMVECs. No interaction was detected between *PHD2* and *HIF2α* silencing (*P* > 0.5 for the interaction; Fig. 2, *E* and *F*). Similar results were also found in PAECs (Supplemental Fig. S2*B*). Therefore, PHD2 silencing similarly induced apoptosis resistance in both the presence and absence of elevated HIF2α protein. To investigate whether HIF1α rather than HIF2α might have mediated apoptosis resistance induced by *PHD2* silencing, *HIF1β*, a constitutive cofactor for the HIFα family, was knocked down with *HIF1β* siRNA (Fig. 2*G*). Although *HIF1β* knockdown alone had no effect on caspase 3/7 activation (Fig. 2*H*) and AV/PI staining (Fig. 2*I*), cosilencing *HIF1β* and *PHD2* enhanced apoptosis resistance (*P* < 0.006 for interaction between *HIF1β* siRNA and *PHD2* siRNA; Fig. 2, *H* and *I*). Collectively, these results demonstrate that although *PHD2* silencing stabilizes HIF2α protein, the apoptosis-resistant phenotype associated with PHD2 deficiency is not mediated by HIF2α or other HIF family members in human LMVECs and PAECs.

### *PHD2* Silencing, Independent of HIF2α, Activates AKT and ERK, Inhibits JNK, and Activates STAT1/3 in LMVECs

**Figure 3.**
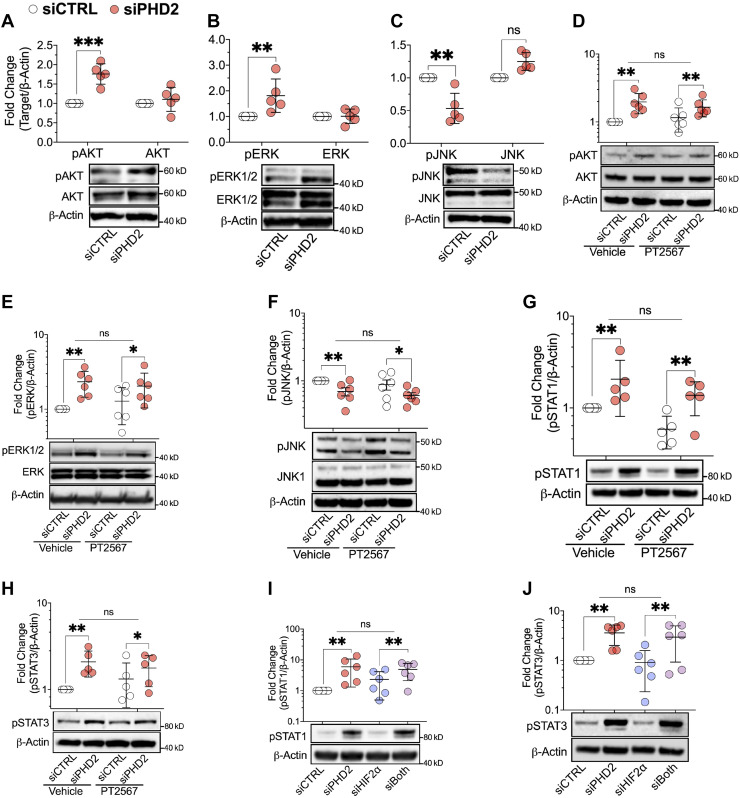
*PHD2* silencing, independent of HIF2α, activates AKT and ERK, inhibits JNK, and activates STAT1/3 in human lung microvascular endothelial cells. *A–C*: cells were transfected with control (siCTRL) or *PHD2* (siPHD2) siRNA for 48 h. Activation of AKT (pAKT-S473), ERK (pERK-T202/Y204), and JNK (pJNK-T183/Y185) were assessed by Western blotting (*n* = 5). *D–H*: cells were transfected as in (*A*) and incubated for another 24 h with HIF2α inhibitor PT2567 (10 µM) or vehicle control. Activation of AKT, ERK, JNK, STAT1 (pSTAT1-Y701), and STAT3 (pSTAT3-Y705 were assessed (*n* = 5 or 6). *I* and *J*: cells were transfected with siCTRL, siPHD2, siHIF2α (HIF2α siRNA), or their combination for 48 h and incubated for another 24 h. Activation of STAT1 and STAT3 were assessed (*n* = 6). Western blot protein levels were normalized to β-actin. Data are presented as means ± SD. **P ≤ 0.01 and ***P ≤ 0.001. Paired *t* test (*A–C*), two-way ANOVA with post hoc *t* test (*D–J*).

Like HIF1α and HIF2α, phosphorylated (activated) AKT can also be directly hydroxylated by PHD2 and subsequently dephosphorylated (inactivated) by VHL-interacting PP2A (19). Thus, besides stabilizing HIF1/2α proteins, loss of PHD2 would be expected to activate AKT. AKT and ERK activation, as well as JNK inhibition, have been associated with apoptosis-resistance (23, 25, 32) and/or IFN pathway activation (23). Therefore, we explored whether *PHD2* silencing affected these kinases in LMVECs. Compared with siCTRL transfected cells, AKT (pAKT-S473; Fig. 3*A*) and ERK (pERK-T202/Y204; Fig. 3*B*) were both activated, and JNK inhibited (pJNK-T183/Y185; Fig. 3*C*) in *PHD2*-silenced LMVECs. Total AKT, ERK, and JNK (Fig. 3, *A*–*C*) were not affected by *PHD2* silencing. Like *PHD2* knockdown, DMOG dose-dependently activated AKT and inhibited JNK (Supplemental Fig. S3, *A* and *B*, respectively). In contrast to *PHD2* silencing, DMOG dose-dependently inhibited ERK activation (Supplemental Fig. S3*C*). Consistent with HIF2α independence, the specific HIF2α inhibitor, PT2567, did not alter AKT and ERK activation, or JNK inactivation in PHD2 deficient LMVECs (Fig. 3, *D*–*F*, respectively).

STAT1 and STAT3 have been implicated in the pathogenesis of PAH (23, 30). STAT1 reflects inflammation, whereas STAT3 promotes cell survival and inhibits apoptosis. Hence, effects of *PHD2* silencing on STAT activation and its HIF2α dependence were also examined in LMVECs. Silencing *PHD2* activated both STAT1 (pSTAT1-Y701; Fig. 3*G*) and STAT3 (pSTAT3-Y705; Fig. 3*H*). Although PT2567 slightly inhibited STAT1 activation (*P* = 0.005 for the main effect of PT2567; Fig. 3*G*), neither the HIF2α inhibitor, PT2567 (Fig. 3, *G* and *H*), nor *HIF2α* silencing (Fig. 3, *I* and *J*) blocked the STAT1 or STAT3 phosphorylation that was induced by PHD2 loss.

### PHD2 Loss Reduces AIP1 Protein Expression and *AIP1* Silencing Mirrors the Signaling and Phenotype Abnormalities Observed in PHD2-Deficient Human LMVECs

**Figure 4.**
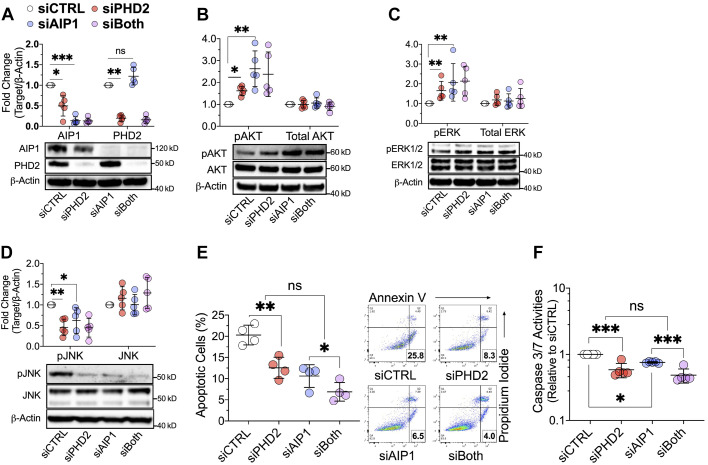
PHD2 loss reduces AIP1 protein expression and *AIP1* silencing activates AKT and ERK, inhibits JNK and induces apoptosis resistance in human lung microvascular endothelial cells. *A–D*: cells were transfected with control (siCTRL), *PHD2* (siPHD2), *AIP1* (siAIP1) siRNA, or their combination for 48 h. AIP1 protein levels and activation of AKT (pAKT-S473), ERK (pERK-T202/Y204), and JNK (pJNK-T183/Y185) were assessed by Western blotting (*n* = 5). *E* and *F*: cells were transfected as in (*A*) and incubated for an additional 24 h in a serum- and growth factor-free medium and then assessed for apoptosis by AV/PI staining and caspase-3/7 assay (*n* = 4 or 5). Western blot protein levels were normalized to β-actin. Data are presented as means ± SD. *P < 0.05, **P ≤ 0.01, and ***P ≤ 0.001. Two-way ANOVA with post hoc *t* test. AV/PI, annexin v/propidium iodide.

AIP1 is a scaffolding Ras-GAP protein, which inhibits both Ras effector pathways, namely, Raf/ERK and PI3K/AKT (24, 33). Furthermore, AIP1 can activate JNK in ECs by de-repressing ASK1 (34). Importantly, endothelial cell-specific *Aip1* knockout has been shown to cause vascular remodeling in mice (35). These previous findings and our present results led us to test whether *PHD2* silencing altered AIP1 expression. Compared with siCTRL transfected cells, AIP1 protein was markedly decreased by *PHD2* silencing (siPHD2) in LMVECs (Fig. 4*A*) while AIP1 mRNA was not affected (Supplemental Fig. S3*D*), consistent with either reduced translation or increased degradation. Like *PHD2* silencing, DMOG dose-dependently decreased AIP1 protein (Supplemental Fig. S3*E*). The specific HIF2α inhibitor PT2567 did not alter AIP1 suppression in PHD2-deficient LMVECs (Supplemental Fig. S3*F*), demonstrating that the effect of PHD2 deficiency on AIP1 is independent of HIF2α. We next investigated the relevance of PHD2 loss-induced suppression of AIP1 to altered AKT, ERK, and JNK activation in LMVECs. *AIP1* silencing (siAIP1) reduced AIP1 protein by 90% and did not affect PHD2 protein levels (Fig. 4*A*). Like *PHD2* silencing, AIP1 knockdown activated AKT (*P* < 0.001 for siAIP1 vs. siCTRL; Fig. 4*B*) and ERK (*P* = 0.002 for siAIP1 vs. siCTRL; Fig. 4*C*), and suppressed JNK activity (*P* = 0.014 for siAIP1 vs. siCTRL; Fig. 4*D*). Apoptosis resistance to serum and growth factor withdrawal was also detected in *AIP1*-silenced LMVECs (*P* < 0.001 and *P* = 0.007 for the main effect of siAIP1, respectively; Fig. 4, *E* and *F*). No interaction was observed between siAIP1 and siPHD2 on apoptosis (*P* > 0.5 for interaction in both; Fig. 4, *E* and *F*).

### Loss of AIP1 Activates STAT1/3, While Inhibition of AKT Reduces STAT1/3 Activation and Apoptosis Resistance in *PHD2*- or *AIP1*-Silenced Human LMVECs

**Figure 5.**
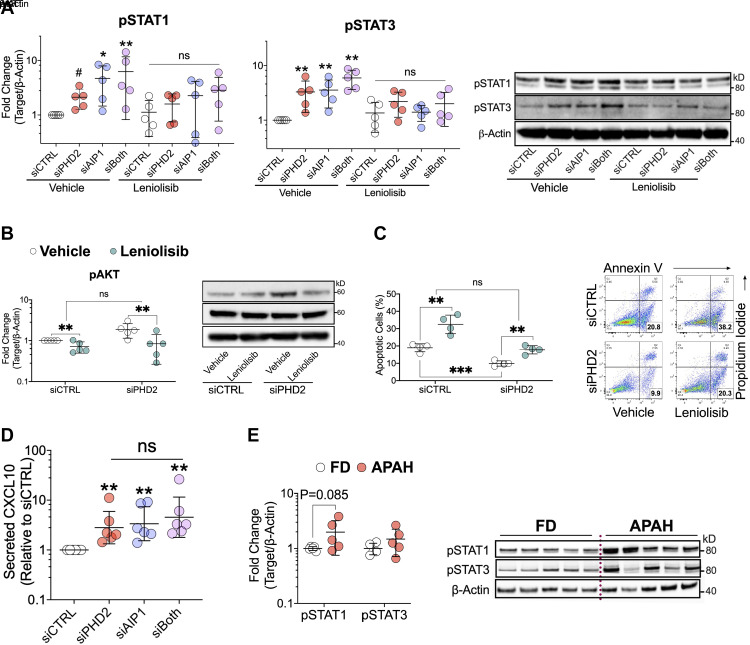
AKT inhibition inhibits STAT activation and reverses apoptosis resistance in *PHD2*- or *AIP1*-silenced human lung microvascular endothelial cells. Cells were transfected with control (siCTRL), *PHD2* (siPHD2), *AIP1* (siAIP1) siRNA, or their combination. *A* and *B*: 48 h posttransfection, cells were further incubated for 24 h in a complete medium with specific PI3Kδ/AKT inhibitor leniolisib (5 µM) or vehicle control. Activation of STAT1 (pSTAT1-Y701), STAT3 (pSTAT3-Y705), and AKT (pAKT-S473) was assessed by Western blotting (*n* = 5). *C*: 48 h posttransfection, cells were further incubated for 24 h in a serum- and growth factor-free medium with leniolisib or vehicle and assessed for apoptosis by AV/PI staining (*n* = 4). *D*: 72 h posttransfection, cell culture supernatants were collected for measuring secreted CXCL10 levels by ELISA (*n* = 6). *E*: lung microvascular endothelial cells isolated from patients with APAH and failed donor (FD) controls were cultured in a complete medium and assessed activation of pSTAT1-Y701 and pSTAT3-Y705 (*n* = 5). Western blot protein levels were normalized to β-actin. Data are presented as means ± SD. #*P* = 0.07; *P < 0.05; **P ≤ 0.01. Two-way ANOVA with post hoc *t* test (*A–D*), unpaired *t* test (*E*). APAH, associated pulmonary arterial hypertension; AV/PI, annexin v/propidium iodide.

AIP1 has been shown to inhibit STAT1/3 in VSMCs, and STAT1 activation in ECs has been ascribed to PI3K/AKT signaling (23, 36). Therefore, we hypothesized that, like PHD2, AIP1 deficiency in LMVECs might also activate IFN/STAT signaling. Silencing *AIP1* alone or in combination with *PHD2* knockdown, significantly activated STAT1/3 in LMVECs (Fig. 5*A*). Leniolisib, a recently FDA-approved specific and well-tolerated PI3Kδ inhibitor (37), blocked STAT1/3 activation (Fig. 5*A*). At the concentration used (5 µM), leniolisib inhibited AKT activation in both siCTRL transfected and *PHD*2-silenced LMVECs (Fig. 5*B*). AKT inhibition promoted apoptosis as measured by AV/PI staining (*P* < 0.001 for leniolisib vs. vehicle within siCTRL; Fig. 5*C*) and caspase 3/7 activation (*P* < 0.001 for leniolisib vs. vehicle within siCTRL; Supplemental Fig. S4) and partly reversed apoptosis resistance induced by *PHD2* silencing (*P* < 0.001 for leniolisib vs. vehicle within siPHD2; Fig. 5*C* and Supplemental Fig. S4).

Consistent with STAT1/3 activation caused by PHD2 or/and AIP1 loss, secretion of CXCL10, an IFNγ signature gene, was induced by both *PHD2* and *AIP1* silencing in LMVECs (Fig. 5*D*). Moreover, LMVECs isolated from patients with APAH with congenital cardiac shunts, often accompanied by hypoxemia that suppresses endothelial PHD2 activity, showed a trend toward increased STAT1/3 activation compared with control LMVECs from FD subjects (*P* = 0.085 for pSTAT1, *P* = 0.036 for the combined pSTAT1 and pSTAT3 effect; Fig. 5*E*). In contrast, ECs derived from patients with IPAH displayed donor-to-donor variability in STAT activation (data not shown), likely reflecting the heterogeneity in IPAH. These results suggest that loss of PHD2 and/or AIP1 in LMVECs leads to activation of the STAT/IFN pathway and that signaling through AKT, independent of HIFα, contributes to this activation.

### Transcriptome Analysis of Human LMVECs after Silencing *PHD2*, *AIP1*, or Both

**Figure 6.**
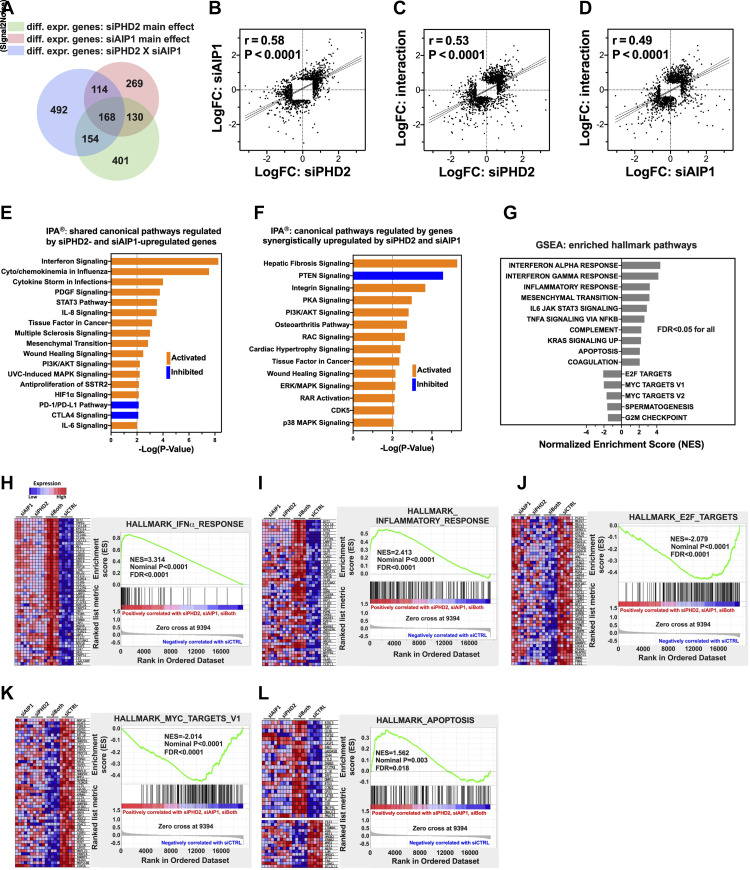
Transcriptome analysis of human lung microvascular endothelial cells silenced for *PHD2*, *AIP1*, and both using microarrays. *A*: Venn diagram of 1,236 genes differentially regulated by silencing *PHD2* (siPHD2) or *AIP1* (siAIP1), 928 synergistically regulated, and their overlaps. *B*: positive correlation between LogFC (fold change) of genes regulated by siPHD2 and siAIP1 (853 and 681 genes, respectively). *C* and *D*: positive correlation between LogFC of synergistically regulated genes and genes regulated by siPHD2 or siAIP1. *E* and *F*: top canonical pathways enriched by ingenuity pathway analysis (IPA, *P* ≤ 0.01) for genes upregulated by siPHD2 and siAIP1 (*E*) and genes synergistically upregulated (*F*). *G–L*: gene set enrichment analysis (GSEA) revealed significant activation of IFNα and inflammation, and suppression of E2F and MYC targets by silencing of PHD2, AIP1, and both (siCTRL vs. siPHD2, siAIP1, and siBoth; FDR < 0.05). This was accompanied by the induction of anti-apoptotic genes (*BIRC3, TAP1,* and *PAK1*) and repression of proapoptotic genes (*BCL2L11, FAS,* and *BTG2*). FDR, false discovery rate.

Transcriptomic profiling of LMVECs using microarrays identified 1,236 genes that were differentially regulated after *PHD2* (siPHD2, 853 genes; Supplemental Table S4) or *AIP1* (siAIP1, 681 genes; Supplemental Table S5) silencing [fold change (FC) ≥ 1.5 and false discovery rate (FDR) ≤ 0.01 for main effects; Fig. 6*A*], and 928 genes (Supplemental Table S6) that were regulated synergistically, either positively or negatively, by *PHD2* and *AIP*1 knockdown [FDR ≤ 0.1 for the interaction between siPHD2 and siAIP1; Fig. 6*A*]. Although only 298 genes (298/1,236 = 24.11%) were shared between siPHD2 and siAIP1 (Fig. 6*A*), log2 fold change (LogFC) of 853 genes differentially regulated by siPHD2 and the LogFC of 681 genes differentially regulated by siAIP1 were positively and significantly correlated (*r* = 0.58, *P* < 0.0001; Fig. 6*B*). The same was true for the LogFC between genes differentially regulated by either siPHD2 or siAIP1 and genes synergistically regulated by *PHD2* and *AIP1* silencing (Fig. 6, *C* and *D*). These results suggest that PHD2 loss and AIP1 loss have overlapping and concordant effects on gene expression in LMVECs through similar effects on specific signaling pathways.

A recent study using single-cell RNA sequencing found that 33 genes were significantly upregulated in the pulmonary ECs of patients with IPAH compared with control subjects (FDR ≤ 0.1) (38). Interestingly, one-third of these genes were also upregulated by *PHD2* silencing in human LMVECs in our study including *IGFBP4* (upregulated by 1.69-fold; FDR = 4.71E-7, see Supplemental Table S7). Notably, elevated serum IGFBP4 levels have recently been linked to PAH and its severity (39). Furthermore, another independent study identified a group of signature genes in pulmonary ECs of patients with PAH, including *TM4SF1* and *PROCR*, which were significantly downregulated and upregulated, respectively (40). Consistent with these findings, siPHD2 significantly upregulated *PROCR* and downregulated *TM4SF1* in LMVECs (see Supplemental Table S7).

Ingenuity pathway analysis (IPA) was used to identify canonical pathways associated with silencing of *PHD2*, *AIP1*, or both, by uploading our selected main effect lists of differentially up- (Fig. 6*E*) and downregulated (Supplemental Fig. S5*A*) genes. Multiple overlapping cellular signaling pathways were found to be significantly activated (Fig. 6*E*) or inhibited (Supplemental Fig. S5*A*) by both *PHD2* and *AIP1* silencing. Among the significantly activated canonical pathways—IFN, cytokine/chemokine, STAT3, PI3K/AKT, and MAPK signaling—IFN and cytokine/chemokine signaling had the most significant *P* values (Fig. 6*E*). Similarly, for genes that were synergistically up- (Fig. 6*F*) and downregulated (Supplemental Fig. S5*B*) by *PHD2* and *AIP1* silencing, IPA identified PI3K/AKT and ERK/MAPK as significantly activated canonical pathways and PTEN signaling as inhibited (Fig. 6*F*). Together, these IPA results support our experimental findings that loss of PHD2 or AIP1 in human LMVECs leads to activation of AKT, ERK, and IFN/STAT.

Likewise, gene set enrichment analysis, another pathway enrichment method that analyzes all genes without arbitrary selection cutoffs, also revealed that gene sets regulating IFNα/γ, inflammation, STAT3, KRAS, and apoptosis signaling were significantly enriched by silencing *PHD2* (siPHD2), *AIP1* (siAIP1), or both (siBoth) compared with control siRNA (siCTRL; Fig. 6*G*). In contrast, targets of E2F and MYC, well-known regulators of cell apoptosis (41, 42), and the G2M checkpoint were significantly enriched in siCTRL (Fig. 6*G*). Expression heat maps of the top 50 marker genes and enrichment plots of these pathways exhibited the activation of IFNα/γ (Fig. 6*H* and Supplemental Fig. S5*C*), inflammation (Fig. 6*I*), and KRAS (Supplemental Fig. S5*D*), the suppression of E2F (Fig. 6*J*) and MYC targets (Fig. 6*K*), and the G2M checkpoint (Supplemental Fig. S5*E*) by silencing *PHD2* or *AIP1*, or both. As is visible in the heat maps, cosilencing had the most pronounced effects on these pathways. Among the enriched apoptosis-related gene sets, silencing of *PHD*2, *AIP1*, or both led to the induction of anti-apoptotic genes like *BIRC3* (*C-IAP2*), *TAP1* (*PSF1*), and *PAK1* while simultaneously repressing pro-apoptotic genes such as *BCL2L11 (BIM)*, *FAS, and BTG2* (Fig. 6*L*). In addition, silencing either *PHD2* or *AIP1* also upregulated other anti-apoptotic genes, including *BCL2L1* (*BCL-XL*) and *MCL1* (*BCL2L3*), both members of the BCL2 family, although these are not shown in the heat map. Together, these results demonstrated that PHD2 loss induced AKT activation and suppressed expression of AIP1 protein, with both effects contributing to an apoptosis-resistant and inflammatory pulmonary vascular endothelial phenotype.

### PHD2 Loss and AIP1 Dysregulation Associated with AKT and STAT3 Activation in Rodent Models of PAH

**Figure 7.**
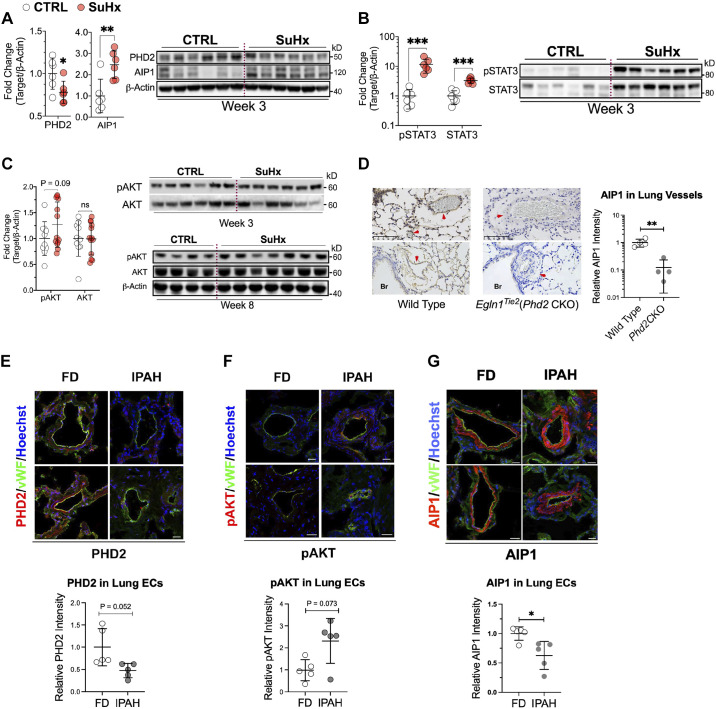
Diminished PHD2, dysregulated AIP1, and activated STAT and AKT in lungs of pulmonary arterial hypertension (PAH) animal models and in lung endothelial cells (ECs) of patients with PAH. *A–C*: PAH was induced in rats using the SU5416/hypoxia protocol (SuHx). Protein levels of PHD2, AIP1, pSTAT3 (Y705), STAT3, pAKT (S473), and AKT in lung tissue were measured at *week 3* and *week 8* compared with normoxic controls (CTRL). Western blot analyses were normalized to β-actin (*n* = 6–13). *D*: mice with conditional *Phd2* knockout [*Egln1^Tie2^*(*Phd2* CKO)] developed PAH. Immunohistochemistry revealed reduced AIP1 expression in the lung vessels of knockout mice compared with wild-type controls (WT). Representative images are shown, and vessels (red arrowheads) and bronchioles (Br) indicated (*n* = 4). Scale bar, 100 µm. *E–G*: lung sections from idiopathic PAH (IPAH) patients and failed donor (FD) controls were costained for von Willebrand factor (vWF, green), PHD2, pAKT (S473) or AIP1 (red), and counterstained with Hoechst 33342 (blue). Representative immunofluorescence images are shown (*n* = 5). Yellow staining identifies the presence of PHD2, pAKT, and AIP1 in lung vascular endothelial cells (ECs). Scale bar: 25 µm. All data are presented as means ± SD. *P < 0.05, **P ≤ 0.01, and ***P ≤ 0.001. Unpaired *t* test.

*PHD2* silencing in LMVECs reduced AIP1, activating AKT and STAT, which contributed to a PAH-like cellular phenotype. Consistent with these in vitro findings, PHD2 protein levels were reduced (Fig. 7*A*), and both phosphorylated (Y705) and total STAT3 levels were increased (Fig. 7*B*) in lung tissue homogenates obtained from SuHx rats 3 wk after PAH model induction. At 3 and 8 wk following model induction, a trend toward increased pAKT (S473) was observed in lung homogenates from SuHx rats relative to normoxia control rats (*P* = 0.09; Fig. 7*C*), whereas total AKT expression was similar.

In contrast to decreased AIP1 protein levels in *PHD2*-silenced LMVECs, whole lung AIP1 expression was increased in SuHx rats (Fig. 7*A*). Western blotting of whole lung tissue from rodent PAH models has limitations, as results can be confounded by the presence of nonendothelial cell types. These include immune cells, VSMCs, and fibroblasts, which are particularly abundant in the inflammatory and proliferative microenvironment of PAH vascular remodeling. Therefore, IHC was utilized to stain target proteins on ECs in the *Egln1^Tie2^*(*Phd2* CKO) mouse model of PAH. IHC staining of lung sections showed that AIP1 (brown staining) in lung vessels of *Egln1^Tie2^*(*Phd2* CKO) PAH mice was significantly reduced compared with control wild-type (WT) mice (Fig. 7*D*). Differences (red arrowheads) in AIP1 staining between WT and PAH mice were seen within the endothelial lining of small pulmonary blood vessels (Fig. 7*D*).

### Diminished PHD2 and Dysregulated AIP1 Associated with AKT Activation in the Pulmonary Endothelium of Patients with PAH

To further address the limitations of whole lung Western blotting, IF staining was performed on lung tissue from patients with IPAH compared with FD control subjects. Consistent with previous reports demonstrating decreased PHD2 (31) and activated AKT (43) in lung vascular ECs from patients with IPAH, costaining for PHD2 or pAKT (S473) and von Willebrand factor (vWF), an endothelial cell marker, demonstrated that PHD2 was diminished (Fig. 7*E*) while pAKT was increased (Fig. 7*F*) in lung ECs of patients with IPAH compared with FD control specimens. AIP1 was highly expressed in the VSMCs of both IPAH and FD control lung tissue (Fig. 7*G*). VSMCs lining remodeled vessels in IPAH displayed stronger AIP1 staining compared with VSMCs of normal arteries in FD controls (Fig. 7*G*). Endothelial cell AIP1 was seen in normal, small lung vessels of FD controls but was largely absent in IPAH (Fig. 7*G*). The increase in AIP1 expression in VSMCs and decrease in ECs, while consistent with elevated AIP1 levels in SuHx rat whole lung tissue and AIP1 loss in *PHD2*-silenced human LMVECs, requires further investigation.

### Mechanism of AIP1 Protein Loss in *PHD2*-Silenced LMVECs

**Figure 8.**
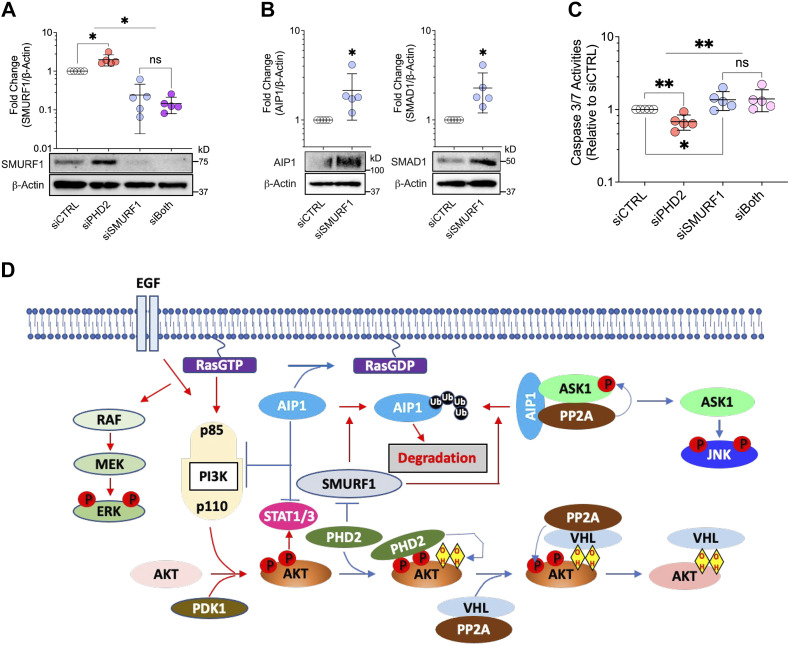
*PHD2* silencing increases SMURF1 while *SMURF1* knockdown increases AIP1 and reverses PHD2 silencing-induced apoptosis resistance in human lung microvascular endothelial cells. Cells were transfected with control (siCTRL), PHD2 (siPHD2), SMURF1 (siSMURF1) siRNA, or both for 48 h. *A* and *B*: whole cell lysates were collected for Western blotting to assess SMURF1, AIP1, and SMAD1 expression. Protein levels were normalized to β-actin, and representative blots were shown. *C*: cells were further incubated for 24 h in a serum- and growth factor-free medium to measure caspase activities using the Caspase-Glo3/7 Assay. Data are presented as means ± SD. *P < 0.05 and **P ≤ 0.01 (*n* = 5 for all). Two-way ANOVA with post hoc *t* test (*A* and *C*), paired *t* test (*B*). *D*: schematic of HIF-independent mechanisms associated with PHD2 loss in pulmonary vascular endothelial cells. The resulting signal transduction (red arrows) and phosphorylation (red circles) events include AKT activation, AIP1 loss, ERK activation, and JNK inhibition, leading to apoptosis resistance and inflammation.

Finally, the E3-ubiquitin ligase SMURF1 (SMAD ubiquitin regulatory factor 1) has been reported to target AIP1 for proteasomal degradation (44). Consistent with this, silencing *PHD2* increased SMURF1 protein expression (Fig. 8*A*) while *SMURF1* silencing increased AIP1 protein levels in LMVECs. Likewise, SMAD1, another known SMURF1 target protein, increased in response to *SMURF1* silencing (Fig. 8*B*). Moreover, *SMURF1* knockdown reversed the apoptosis-resistant phenotype of *PHD2*-silenced LMVECs (Fig. 8*C*). These results implicate SMURF1 in *PHD2* knockdown induced AIP1 loss and the pseudohypoxic, endothelial phenotype described here.

## DISCUSSION

In this study, we demonstrated that PHD2 deficiency leads to an apoptosis-resistant and inflammatory endothelial cell phenotype independent of HIF signaling. Mechanistically, as summarized in the schematic Fig. 8*D*, PHD2 deficiency activated AKT and reduced AIP1 protein expression. Decreased AIP1 in turn contributed to AKT and ERK activation and JNK inactivation, which was then linked back to an apoptosis-resistant and inflammatory phenotype that has been associated with vascular remodeling in PAH.

Inherited PHD2 loss-of-function mutations, P317R, H374R, and D254H, caused erythrocytosis due to stabilized HIF2α in six affected patients but have not yet been reported to cause either PH or PAH (45–47). Similarly, eight separate cases of erythrocytosis linked to novel hereditary heterozygous HIF2α, gain-of-function mutations, M535V/I, G537W, and D539E, did not present with PAH or PH (48–50). Cellular transcriptomic profiles have revealed that numerous genes undergo differential expression in response to hypoxia or pseudohypoxia, yet remain unaffected by HIF silencing (51). A recent study of pulmonary arteries from patients with PH revealed elevated levels of HIF proteins, increased cytokines/chemokines, and upregulation of anti-apoptosis genes (52). However, inhibiting HIF did not reverse the expression of these genes or the aberrant phenotype of fibroblasts cultured ex vivo (52). Likewise, in our experiments, silencing or inhibiting PHD2 in human LMVECs and PAECs resulted in resistance to apoptosis and an inflammatory state, but blocking HIF signaling did not reverse these effects. On the contrary, *HIF2α* or *HIF1β* silencing somewhat increased apoptosis resistance. These findings collectively support the existence of pathways beyond HIFα genomic signaling that mediate crucial aspects of hypoxia, pseudohypoxia, and PH or PAH.

As already noted here and by others (19, 53), PHD2 loss or its inhibition by small molecule inhibitors or hypoxia leads to HIF-independent AKT activation. PHD2 activation via its cofactor α-ketoglutarate inactivates AKT and rescues mice from lung inflammation (53). Consistent with these findings, *PHD2* silencing or inhibition by DMOG in LMVECs induced AKT activation and triggered an IFN-biased inflammatory response. Importantly, these effects of *PHD2* silencing in the present study were not altered by *HIF2α* siRNA silencing or PT2567, a selective HIF2α inhibitor. In a similar HIF2α-independent manner, *PHD2* silencing in LMVECs activated ERK while suppressing JNK and reducing AIP1 protein levels without altering AIP1 mRNA expression. Likewise, DMOG mimicked all these effects of *PHD2* silencing except for ERK phosphorylation, indicating that *PHD2* silencing and its inhibition by DMOG are not entirely equivalent.

As a scaffolding Ras GAP, AIP1 can directly interact with AKT1 and the p85 subunit of PI3K, thereby inhibiting the PI3K-AKT axis (33). AIP1 GAP activity further enforces this inhibition by reducing RAS-dependent activation of PI3K p100α subunit (24) and negatively regulating ERK (54). Binding both ASK1 and PP2A, AIP1 facilitates the removal of the inhibitory ASK1-S967 phosphorylation and accordingly activates the ASK1-JNK pathway (34). Loss of AIP1 thereby suppresses JNK activation and JNK-mediated apoptosis while also activating IFN/STAT signaling (36). Consistent with these previous results, *AIP1* silencing, like PHD2, activated AKT and ERK, inhibited JNK, and activated STAT1/3 in our study. Cosilencing showed pronounced, concordant effects, as revealed by microarray analysis. STAT1/3 activation was also detected in LMVECs isolated from patients with APAH. These abnormalities were accompanied by apoptosis resistance in LMVECs, suggesting that AIP1 loss in PHD2-deficient ECs contributes to AKT, ERK, and STAT activation, JNK suppression, and subsequent cellular phenotypic aberrations.

AKT activation has been found in the vasculopathic pulmonary vessels of hypoxic mice (55), SuHx rats (56, 57), and patients with IPAH (43, 56). Deletion of *Akt1* (55) or pharmacological inhibition of PI3K, an upstream AKT activator, attenuates vascular remodeling and the development of PAH in rodents (56). Moreover, AKT and STAT1/3 activation was recently demonstrated in dermal fibroblasts from patients with HPAH due to CAV1 loss-of-function mutations (23). Consistent with these prior findings, the present study showed augmented activation of AKT and STAT3 along with reduced PHD2 levels in lung homogenates of SuHx rats, supporting the link between PHD2 deficiency and increased AKT and STAT activity in PAH. Notably, the importance of AKT signaling in STAT activation has also been reported by others. STAT1 requires phosphorylation at tyrosine 701 and serine 727 for full activation. AKT directly phosphorylates STAT1 at serine 727 (58) and IRF3 at serine 386 (59). Phosphorylated IRF3 induces expression of IFNs, which activates JAK with subsequent phosphorylation of STAT1 at tyrosine 701 and STAT3 at tyrosine 705 (59). In addition, AKT reinforces STAT signaling by promoting mRNA translation of IFN-stimulated genes via phosphorylation of mTOR/p70 S6 kinase (60).

AIP1 is expressed in both ECs and VSMCs. Mice with global deletion of *Aip1* exhibit enhanced inflammation and endothelial dysfunction induced by hyperlipidemia (61). ECs isolated from *Aip1*-knockout mice or *AIP1*-silenced human ECs all show attenuated proliferation (62), but VSMCs in endothelial cell-specific *Aip1* knockout mice are highly proliferative due to AIP1 loss-induced endothelial inflammation (35). Like *PHD2*-silenced LMVECs, AIP1 protein levels assessed by immunohistochemistry were markedly decreased in the pulmonary endothelium of *Tie2-Cre*/*Phd2* knockout mice. In contrast, lung homogenates from SuHx rats, which had decreased PHD2 expression, were found to have increased levels of AIP1. This apparent paradox might be explained by the cross talk between endothelium and its underlying vascular smooth muscle. Although endothelial PHD2 deficiency suppresses AIP1 expression in these cells, the resulting inflammatory endothelium may induce VSMC hyperproliferation and AIP1 expression. Further investigation is warranted to clarify these possible cell-cell interactions in future studies. Notably, PHD2 deficiency caused a reduction in AIP1 protein but not mRNA in human LMVECs, suggesting posttranslational regulation of AIP1 by PHD2. SMURF1, an E3-ubiquitin ligase, can target AIP1 for proteasomal degradation, which is enhanced by AKT phosphorylation of SMURF1 and AIP1 (44, 63). Importantly, SMURF1 is increased in the tissues of patients with PAH and *Smurf1* deletion protects mice from PAH (64), indicating a critical role of SMURF1 and its degradation targets in disease development. Consistent with these previous findings, silencing *PHD2* increased SMURF1 protein, whereas knocking down *SMURF1* increased AIP1 protein in LMVECs. Moreover, knocking down *SMURF1* reversed the apoptosis-resistant phenotype of *PHD2*-silenced cells.

It is important to acknowledge that our in vitro model using PHD2-silenced pulmonary ECs cannot fully replicate the complex effects and underlying mechanisms of PHD2 deficiency in a heterogeneous and multifaceted disease like PAH, which affects various cell types in the pulmonary vasculature. Further studies using isolated lung ECs and VSMCs from patients with PAH, and the SuHx rat model of PAH, as well as other hypoxia-induced and genetic rodent models, are needed to provide additional support for our findings. Ultimately, the development of successful treatment strategies targeting the mechanisms reported here would be the best evidence supporting their importance in the pathobiology of PAH.

In summary, pseudohypoxia simulated by PHD2 deficiency in human lung ECs induces an apoptosis-resistant and inflammatory phenotype. Investigation of this in vitro cellular model revealed that AKT and STAT activation, as well as AIP1 loss, but not HIF signaling, were critical to these abnormalities. Two different rodent models of PAH and lung tissue from patients with PAH supported the HIFα-independent connection between PHD2 loss and the activation of AKT and STAT signaling. At least in part, this aberrant signaling and dysfunctional EC phenotype was attributable to SMURF1-mediated degradation of AIP1, a scaffolding Ras GAP that regulates multiple intracellular signal transduction pathways. PHD2, AKT, STAT 1/3, and AIP1 are important regulatory nodes in the endothelial dysfunction of hypoxia, pseudohypoxia, and PAH.

## DATA AVAILABILITY

Data will be made available upon reasonable request.

## SUPPLEMENTAL MATERIAL

10.6084/m9.figshare.26306104 Supplemental Figs. S1–S6 and Supplemental Tables S1–S7: https://doi.org/10.6084/m9.figshare.26306104.

## GRANTS

This work was supported by intramural funds from the National Institutes of Health Clinical Center.

## DISCLOSURES

No conflicts of interest, financial or otherwise, are declared by the authors.

## AUTHOR CONTRIBUTIONS

S.W. and R.L.D. conceived and designed research; S.W., K.S.A., L.-Y.C., M.A.H.S., G.A.F., C.L.W., T.J., Z.-X.Y., K.T., and Y.-Y.Z. performed experiments; S.W. and C.Y.D. analyzed data; S.W., K.S.A., M.A.H.S., Z.-X.Y., K.T., J.M.E., and R.L.D. interpreted results of experiments; S.W. and L.-Y.C. prepared figures; S.W. drafted manuscript; S.W., K.S.A., L.-Y.C., G.A.F., Y.-Y.Z., J.M.E., and R.L.D. edited and revised manuscript; S.W., K.S.A., L.-Y.C., M.A.H.S., G.A.F., C.L.W., T.J., Z.-X.Y., K.T., C.Y.D., Y.-Y.Z., J.M.E., and R.L.D. approved final version of manuscript.
